# “R” you getting this? Factors contributing to the public’s understanding, evaluation, and use of basic reproduction numbers for infectious diseases

**DOI:** 10.1186/s12889-024-18669-6

**Published:** 2024-05-01

**Authors:** Ruben D. Vromans, Nadine Bol, Marloes M. C. van Wezel, Emiel J. Krahmer

**Affiliations:** Department of Communication and Cognition, Tilburg center for Cognition and Communication, Tilburg School of Humanities and Digital Sciences, Tilburg University, P.O. Box 90153, Tilburg, 5037 LE The Netherlands

**Keywords:** COVID-19 Reproduction number, Evaluative labels, Infectious diseases, Numeracy, Risk communication, Visual aids

## Abstract

**Background:**

We (1) examined the effects of evaluative labels and visual aids on people’s understanding, evaluation, and use of the COVID-19 reproduction number (or “r-number”), (2) examined whether people’s perceived susceptibility and (intended) adherence to preventive measures changed after being exposed to the r-number, and (3) explored whether these effects and changes depended on people’s numeracy skills.

**Methods:**

In an online experiment, participants from a large Dutch representative sample (*N* = 1,168) received information about the COVID-19 r-number displayed on the corona dashboard of the Dutch Ministry of Health, Welfare and Sport. The r-number was either presented with or without a categorical line display (i.e., evaluative label) and with or without an icon-based tree diagram (i.e., visual aid) explaining how the number works. Regarding people’s use of the statistic, we measured perceived susceptibility to COVID-19 and adherence (intention) to five preventive measures before and after exposure to the r-number. After exposure, we also measured participants’ understanding, perceived usefulness, affective and cognitive evaluation, and objective numeracy.

**Results:**

About 56% of participants correctly interpreted the r-number, with highly numerate people having better understanding than less numerate people. Information about the r-number was perceived as more useful when presented with a visual aid. There were no differences across experimental conditions in people’s understanding, affective, and cognitive evaluations. Finally, independent of experimental conditions, intention to adhere to preventive measures was higher after seeing the r-number, but only among highly numerate people.

**Conclusions:**

Although evaluative labels and visual aids did not facilitate people’s understanding and evaluation of the r-number, our results show that the statistic is perceived as useful and may be used to stimulate adherence to preventive measures. Policy makers and public health communicators are advised to clearly explain why they are giving these numbers to – especially – the less numerate people, but also how people could use them for behavior change to combat the spread of virus during a pandemic.

## Background

During major infectious disease pandemics, such as influenza and coronavirus diseases (e.g., COVID-19), the general public is usually being bombarded with complex numerical concepts [1, 2]. For instance, during the COVID-19 pandemic, people received numerical information about daily infected cases, patients admitted to the hospitals and intensive cares, or risks of vaccine side-effects. People were also confronted with complex concepts such as uncertainties around scientific evidence, statistical predictions (e.g., about the spread of the virus obtained through advanced statistical models), and exponential growth. In the era of “big health data” [3, 4], policy makers, healthcare professionals, and the general public (including patients) have access to many different types of these health and scientific data related to pandemic outbreaks, for instance via publicly available web-based dashboards [5–7].

A prominent statistic that is frequently being shared with the general public is the reproduction number [1]. This number, also denoted as “r” (or “r-number”), refers to the average number of people that are expected to be infected by one unique individual (e.g., with an “r” of 2, a patient with a certain disease is likely to infect two other people, who in turn are likely to infect two other people, etc.) [8]. An “r” higher than 1 thus means that the number of cases is increasing. It is considered an important statistic, as experts use it to predict how far and fast a virus might spread or to inform policy makers about implementing any preventive measures for containing an outbreak [1]. Public communication of the r-number (and other numerical data) via web-based dashboards may increase risk perceptions and stimulate people to subsequently adopt protective behaviors, such as practicing physical distancing or wearing face masks, which is key in limiting the spread of the virus [9, 10]. Although it is important to calculate the r-number and subsequently share it with the general public for changing their risk perceptions and promoting protective behaviors, we first need people to understand and correctly evaluate the number. That is, the number should not be overly complex or frightening, and it should be perceived as useful in decision-making. Only when people understand and make sense of the numerical information, they can effectively act upon it (i.e., knowing what to do with it) [11].

The aim of this experimental study was threefold. First, we tested whether communicating the COVID-19 r-number with (vs. without) evaluative labels (i.e., categorical line display) and with (vs. without) visual aids (i.e., icon-based tree diagram explaining the r-number) influences people’s understanding and evaluation of the r-number (i.e., affective evaluations, cognitive evaluations, and perceived usefulness). Second, regarding people’s use of the statistics, we examined whether—compared to baseline measurements—perceived susceptibility of the risk of getting COVID-19 and intention to adhere to preventive measures differed after exposure to the r-number. Third, we explored whether these effects and changes differed between less and highly numerate people, thereby relying on a large and representative sample of the Dutch population.

### Challenges with correctly understanding, evaluating, and using the r-number

Despite their relevance and wide implementation, there are several challenges involved with correctly understanding, evaluating, and using r-numbers. For instance, it may be difficult to imagine how the r-number actually works. Typically, only numerical information is being provided about the statistic (e.g., “the r is 1.30”), but it can be challenging to cognitively evaluate the number (i.e., ease of processing) or to visually imagine how such a number can be translated into an exponential growth of more infected cases in society [12, 13]. This lack of understanding of exponential growth in the general public is problematic, as it may lower awareness of epidemic exponential growth, disease-related risk perceptions, and adhering to preventive measures for eventually preventing the virus from spreading exponentially.

Furthermore, to some individuals, the r-number has low “evaluability”, a concept in risk communication that refers to the extent to which people can derive meaning from novel information they receive (e.g., whether they can determine if the r-number is alarming or not) [14, 15]. In this particular case, the r-number is an unfamiliar number and lacks inherent meaning, which makes it extremely difficult for people to evaluate the goodness and the badness of the number, and also its usefulness. As a consequence, this lack of meaning, usefulness, and evaluability of the number means that people will probably not use the number in subsequent decision-making of adopting health-related protective behaviors [16].

This lack of understanding, evaluability, and use also relates to the challenge with not everyone being able to work with numbers and mathematical concepts, also known as “numeracy” [17]. Compared to the highly numerate, less numerate individuals are more likely to avoid and pay less attention to numeric information, have more problems with ignoring irrelevant information or correctly interpreting probabilities, and are less sensitive to numeric information (i.e., the “feel” of numbers) [17–19]. Less numerate people also find it generally hard to derive affective meaning from unfamiliar numerical information, which greatly impacts its usefulness in decision-making as it can be difficult to interpret [11]. As such, more systematic research is needed to determine whether and how understanding, evaluability, and use of the r-number can be increased among the general public with varying numeric abilities, especially in times of health crises and infectious disease outbreaks.

### Factors contributing to increased understanding, evaluation, and use of the r-number

Relying on a profound amount of empirical work in risk communication and cognitive psychology [20–24], multiple ways have been proposed to improve understanding, evaluability, and use of unfamiliar statistics, such as the r-number, also among people with various numeric abilities, ranging from visual support to written narratives. We will focus on two communication strategies: the use of a visual aid for explaining how the r-number “works” (i.e., icon-based tree diagram) and the use of a visual evaluative label (i.e., categorical line display) for increasing the evaluability of the r-number. To better understand the impact of these communication strategies for a wide variety of individuals, we also explore the role of numeracy in the effectiveness of these communication strategies.

#### Visual aids for understanding exponential growth

During the COVID-19 pandemic, visual aids had become increasingly popular and useful for capturing attention and disseminating crucial information about the preventive measures and spread and containment of the virus [25]. From a dual coding perspective, the combination of verbal (e.g., written information such as text or numbers) and visual (e.g., illustrations or visual representations of data) modes of communication positively influences people’s information processing of such complex health-related information [1, 26]. Combining verbal with visual modes can attract more attention, and therefore increases the likelihood of the information being understood [27]. Moreover, following Mayer’s cognitive theory on multimedia learning, illustrations facilitate the creation of mental representations, which facilitates learning [28, 29]. This could especially be useful for processing unfamiliar statistics such as the r-number, which also requires cognitive capacity and creating mental representations for visually imaging how exponential growth works.

Consistent with dual coding theory and CTML, there is large body of literature showing that different types of visual aids can improve understanding and perception of different types of (complex) health statistics [21, 22, 30]. For instance, icon arrays (where stick figures displayed in different colors represent individuals with or without experiencing an event) have been shown to facilitate understanding of various health statistics such as event rates, part-whole relationships, comparative risks, and incremental risks [19, 31, 32]. By presenting health risks as population figures, it has been demonstrated that people can process risks more rapidly and automatically with less cognitive effort. Another type of visual aid, tree diagrams (or natural frequency trees), has been shown to facilitate Bayesian reasoning (i.e., statistical approach that involves updating beliefs or probabilities based on new evidence) and understanding of more difficult numbers such as conditional probabilities [24, 33, 34]. Tree diagrams are designed for intuitive reading and processing, thereby facilitating individuals in breaking down complex mathematical problems (e.g., working with test specificity/sensitivity statistics or absolute/relative statistics) into distinct and manageable steps [34]. While the concept of the r-number may not appear as mathematically complex initially, understanding how many other people can get infected by a particular r-value requires at least some cognitive effort and knowledge about exponential growth. In such cases, a tree diagram is as a promising visual aid to enhance comprehension.

Based on dual coding theory and CTML, as well as prior empirical research, communicating the r-number with both a textual and visual explanation, such as a icon-based tree diagram displaying its functioning, could facilitate people’s objective understanding and their cognitive evaluation of the information (i.e., being easier to process and comprehend). Moreover, one could expect that people perceive the visual explanation of the r-number as useful and increase their time in assessing the information, resulting in an increased likelihood of people increasing their COVID-19 risk perceptions and intentions to better adhere to preventive measures for limiting the spread of the virus.

#### Evaluative labels for increasing evaluation and use

In addition to understanding, numeric information varies in how easy it is to evaluate and use for people. To some, it is straightforward to judge how good or bad the current r-number is, while others may struggle to make sense of the statistic. Indeed, evaluability theory [35, 36] posits that adding certain textual, numerical, or visual information helps people put unfamiliar statistics in context, which increases the evaluability and usage of the numerical information. Put differently, the more evaluable a health statistic is, the better individuals can determine whether it is good or bad and whether they should take action or not. Several experimental studies have shown that when receiving personalized risk information, adding other relevant comparative information such as the average person’s risk may help individuals to better evaluate or estimate their personalized risk [19, 37]. However, telling people whether they are below or above average may also (undesirably) influence their attitudes towards risks and benefits of treatment options [38].

Another way of increasing the evaluability of unfamiliar health statistics is by using textual or visual evaluative labels. For instance, textual interpretative labels can serve different purposes such as describing the magnitude of the risk (e.g., “this is a low risk”) or the severity of the consequences (e.g., “this is good”) [39]. In one study, evaluative labels such as “poor” or “excellent” improved people’s decision-making about the quality of healthcare services [18]. Furthermore, visual evaluative labels such as line displays showing different risk categories, action thresholds, or out-of-range statistics have been shown to improve evaluability and decision-making [14, 15, 18]. A series of experimental studies by Zikmund-Fisher and colleagues showed that such visual labels can help people derive meaning from unknown medical test results and statistics [40, 41]. As such, visual evaluative labels could serve as a potential persuasive tool for changing people’s risk perceptions and behavioral intentions compared to the r-number alone [42]. Given that prior experimental studies have typically focused on the effects of evaluative labels on people’s understanding of individualized health risks, it is interesting to find out how people will evaluate and respond to labels that are added to “societal” quantitative information such as the r-number.

However, like numbers, (visual) evaluative labels are vulnerable to wide variations in their perceived magnitude and may unnecessarily lead to higher affective evaluations or emotional responses due to their categorical nature (e.g., good vs. bad news) [19, 43]. In line with the affect heuristic and traditional dual process models on information processing and persuasion such as the elaboration likelihood model [44, 45], people make risk judgments based on not only their cognitions and rational analyses but also on their affective feelings and heuristic cues. Indeed, visual evaluative labels like colored categorical line displays could potentially elicit greater affective responses through automatic associations (e.g., red is typically associated with danger) compared to numerical information alone, which in turn impacts people’s risk perceptions and affective evaluation of the information [46]. While some individuals may struggle to objectively grasp the significance of the r-number, assigning an evaluative label to this statistic could facilitate rapid heuristic processing of information. This, in turn, could aid individuals in translating unfamiliar numerical data into meaningful actions.

Taken together, we examine whether communicating the r-number with a visual evaluative label would also lead to more increased cognitive and affective evaluations and perceptions of usefulness, and whether the label would help increase people’s COVID-19 risk perceptions and intentions to adhere to preventive measures.

#### Numeracy as moderating factor

We also evaluated the extent to which people’s objective numeracy would moderate these effects and changes. Generally speaking, highly numerate individuals typically have a higher need for numeric information, pay more attention to numeric information and ignore irrelevant information, have a better recall of numeric information, have a better feeling with numbers (i.e., sensitivity to numbers), and are better able to derive affective and evaluative meaning from numeric information, compared to the less numerate [17, 47]. This would suggest that highly numerate individuals would be able to know how to understand, evaluate and use the r-number, regardless of whether it is communicated with visual information or evaluative labels. Less numerate people, on the other hand, struggle more with deriving the core bottom-line meanings about unfamiliar health statistics, and may therefore benefit from visual aids (for explaining how exponential growth works with the r-number) and visual evaluative labels (for telling them whether something should be done with the r-number) [17, 18, 39]. Indeed, in one experimental study, it was found that highlighting the meaning of the most important numeric information (compared to less important information) helped especially less numerate individuals to make better health-related decisions [48]. We therefore address the question to what extent numeracy moderates the effects of visual aids and evaluative labels on people’s understanding and evaluation of the r-number, and their changes in perceived COVID-19 susceptibility and adherence to preventive measures after exposure to the r-number.

## Method

### Design and participants

We used a 2 × 2 × 2 between-subject design, with evaluative label (categorical line display present vs. absent), visual aid (icon-based tree diagram present vs. absent), and objective numeracy (less vs. highly numerate) as independent variables. Before and after the exposure to the r-number during the experiment, we measured participants’ perceived susceptibility of COVID-19 and their (intentions to) adherence scores to several preventive measures. After exposure, we also measured participants’ understanding of the COVID-19 r-number, their affective/cognitive evaluation of the number, and its perceived usefulness. Data collection took place in October 2020, when the number of infected cases with COVID-19 was rising again in the Netherlands. The complete online experiment with materials and measure items and associated dataset can be found at the Open Science Repository [49].

A representative sample of the Dutch population (age ≥ 18) was recruited through CentERdata’s Longitudinal Internet Studies for the Social Sciences (LISS) panel. This panel consists of 5,000 households in the Netherlands, comprising approximately 7,500 individuals, and represents a true probability sample of households drawn from the population register by Statistics Netherlands [50]. Households that could not otherwise participate in the panel are provided with a computer and Internet connection. Panel members complete online questionnaires every month for which they receive financial compensation. In addition, the LISS panel yearly collects data on panel members’ sociodemographics, which were added to the experimental dataset of the current study.

### Materials

**Fig. 1:**
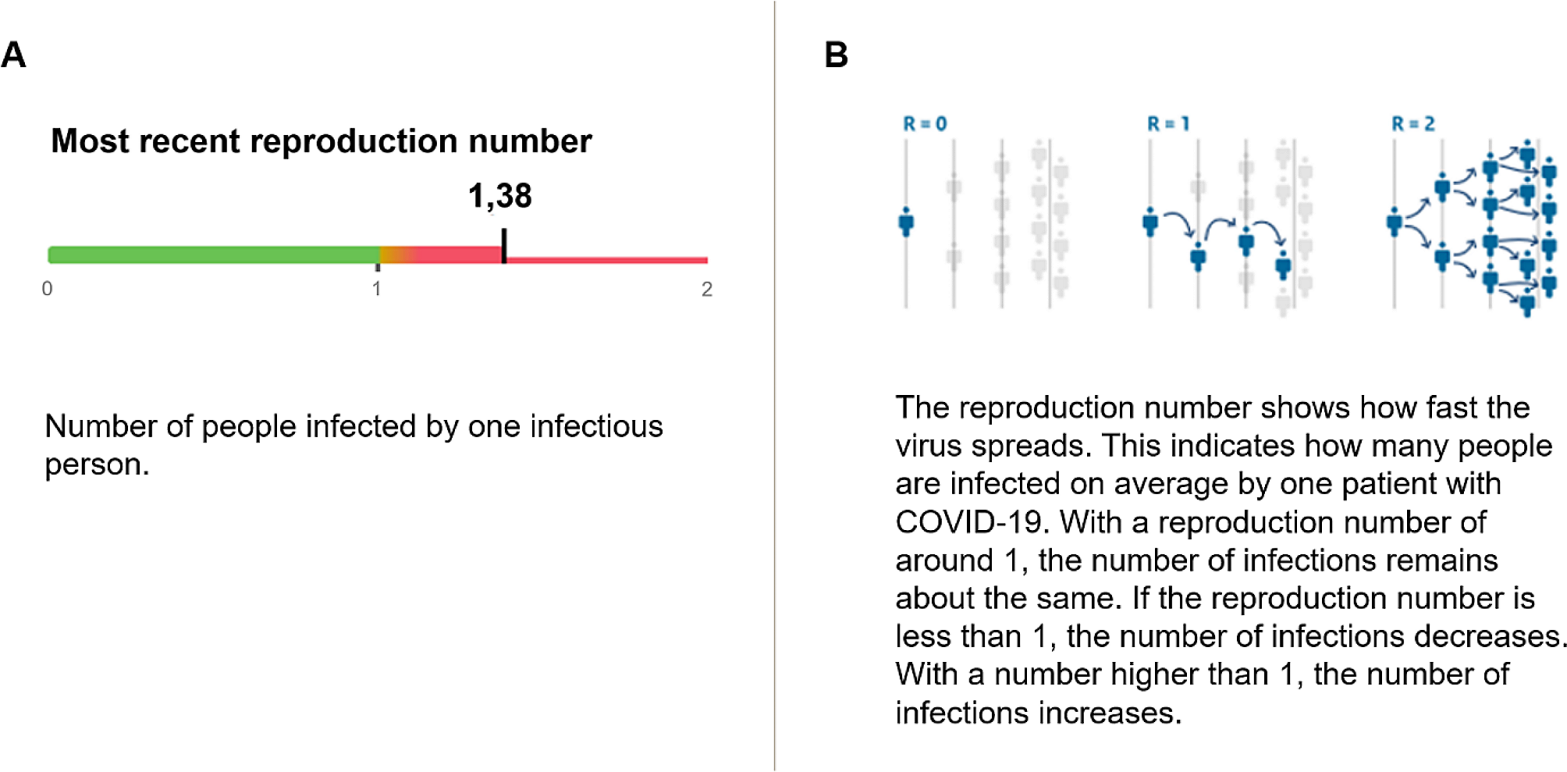
Experimental stimuli consisting of (**A**) an evaluative label using a categorical line display around the actual COVID-19 r-number, and (**B**) a visual aid displaying how the virus with different r-numbers might spread from person to person over three reproductive stages using icon-based tree diagrams

Participants received information about the actual COVID-19 r-number on September 11, 2020 (*r* = 1.38) as originally displayed on the corona dashboard of the Dutch Ministry of Health, Welfare and Sport [51] (for a study on the development of the dashboard, see [5]). In all four experimental conditions, the information consisted of the r-number itself together with the caption “Number of people infected by one infectious person”, and a written explanation of the r-number. Participants either received the r-number with or without an evaluative label, which was operationalized as a categorical line display (see Fig. 1A), which was based on prior work on evaluability by Zikmund-Fisher [15]. This line display had three numbers (from left to right: 0, 1, and 2) and three colors: green (for an r-number between 0 and 1), orange (for an r-number between 1 and 1.1) and red (for an r-number between 1.1 and 2). Furthermore, participants either received the textual explanation of the r-number with or without a visual aid (Fig. 1B), which consisted of an icon-based tree diagram displaying how the virus with a basic r-number of 2 might spread from person to person over three reproductive stages. The evaluative label and the visual aid were both part of the corona dashboard.

### Measures

#### Perceived susceptibility of COVID-19

Before and after exposure to the r-number in the experiment, we measured participants’ perceived susceptibility to COVID-19, referring to people’s beliefs about their chances of getting COVID-19 (four items, α_pre_ = 0.86, α_post_ = 0.87, example item: “I have the feeling that I cannot avoid getting the coronavirus”), measured on a 5-point scale with 1 as “strongly disagree” and 5 as “strongly agree” [52, 53].

#### Adherence and intention to adhere to preventive measures

Adherence to the preventive measures of the National Institute for Public Health and the Environment (RIVM) was measured with five items, using a 5-point scale with 1 as “never” and 5 as “always” [54]. Items included five preventive measures aimed at limiting the spread of the virus: “wash your hands often”, “cough and sneeze into your elbow”, “use paper tissues to blow your nose and discard them after use”, “do not shake hands”, and “keep 1.5 meters (2 arms lengths) distance from other people”. Before exposure to the r-number, we asked to what extent participants adhered to these five preventive measures in the past seven days (α_pre_ = 0.69), while after exposure to the r-number we asked their intention to adhere to these five measures in the upcoming month (α_post_ = 0.77).

#### Objective understanding of the r-number

Objective understanding was measured by asking participants “how many new people would be infected by 100 people who are carrying the coronavirus based on the current reproduction number of 1.38?” (correct answer: 138 people). Participants could enter their response in an open-ended box. Given that our item asked for a precise and accurate answer (instead of a rough estimate), we only counted “138” as a correct response and all other responses as incorrect. Objective understanding was then compressed into a dichotomous variable (i.e., correct vs. incorrect response).

#### Evaluation of the r-number

*Affective evaluation* of the r-number was measured with three items (“How frightening/worrisome/serious do you think the information on the corona dashboard was?”, α = 0.85) that could be rated on a 5-point scale semantic differential scale, with 1 as “not frightening/worrisome/serious” and 5 as “very frightening/worrisome/serious” [43]. *Cognitive evaluation* was also measured with three items (“The information I just saw on the corona dashboard takes me a lot of time and energy to understand/is simple/is clear and easy to understand”, α = 0.81), that could be rated on a 5-point scale with 1 as “strongly disagree” and 5 as “strongly agree”. Finally, *perceived usefulness* was measured with three items (“The information I just saw on the corona dashboard is useful/gives insight into how fast the virus is spreading/helps me to contribute to the fight against the corona virus”, α = 0.69) and were rated on a 5-point scale, with 1 as “strongly disagree” and 5 as “strongly agree” [55, 56].

#### Numeracy

We measured objective numeracy with an existing scale developed by Schwartz and colleagues [57], consisting of three open-ended mathematical questions (e.g., "In a lottery, the chance of winning a 10-euro prize is 1%. 1,000 people buy 1 ticket each for this lottery. How many people will win this 10-euro prize?", α = 0.62). Participants could enter their response in an open-ended box. Each answer was coded as 1 (correct) or 0 (incorrect). Scores of all three questions answered correctly were considered as highly numerate, and all other scores as less numerate.

### Statistical analyses

Associations between the evaluative labels and visual aids on the one hand, and objective understanding on the other were assessed using separate chi-square analyses (aim 1). The role of numeracy was assessed using separate chi-square analyses for less and highly numerate people (aim 3). To assess the effects of evaluative labels, visual aids, and numeracy on people’s evaluations, we fit three analysis of variance (ANOVA) models with affective evaluations, cognitive evaluations, and perceived usefulness as dependent variables, and evaluative labels, visual aid, and numeracy as independent variables (aim 1 and 3). Finally, changes in risk perception and adherence were tested using two mixed-model ANOVAs, with perceived susceptibility of COVID-19 and adherence to preventive measures as dependent variables, and with independent variables consisting of measurement (before vs. after exposure of the r-number) as within-subjects factor, and evaluative label, visual aids, and numeracy as between-subjects factors (aim 2). We also ran these mixed-model ANOVAs for each preventive measure separately, for which we applied a Bonferroni correction (α *=* 0.05/5 = 0.01). Data were entered and analyzed in SPSS version 27.0 (IBM Corporation, Somers, NY, USA).

## Results

### Sample characteristics

**Fig. 2:**
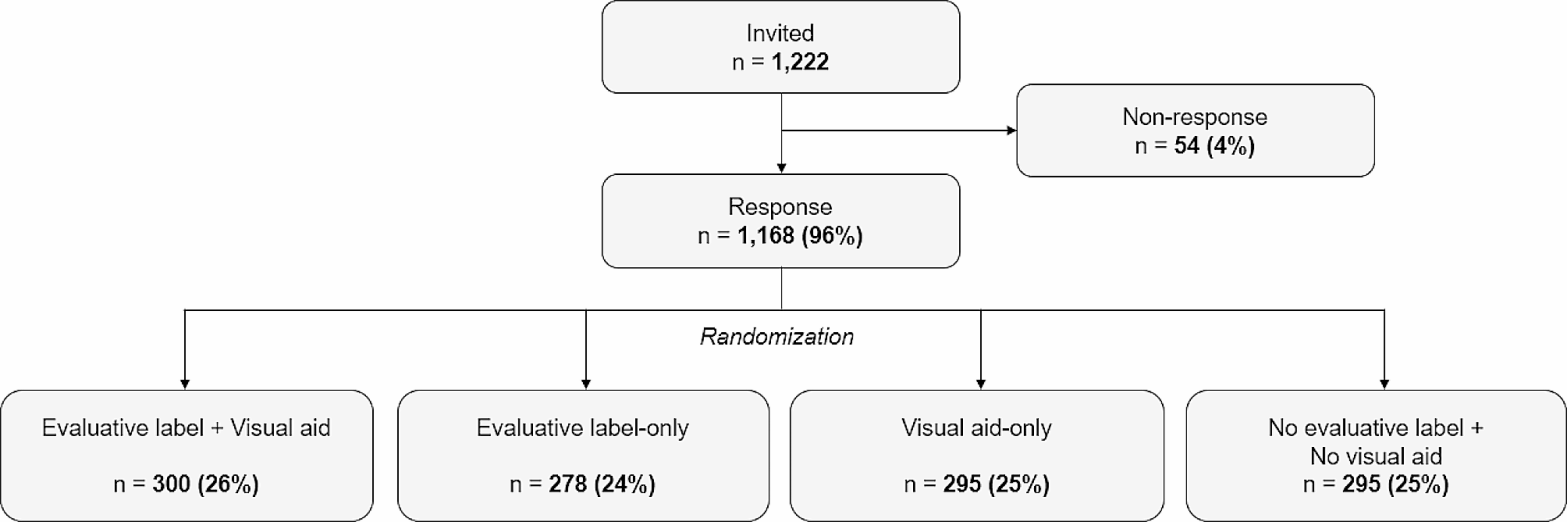
Flowchart of the data collection process

Out of 1,222 people who were invited to participate, 1,168 (96%) clicked on the link to launch the survey (Fig. 2). The mean age of the sample was 55.6 years (*SD* = 17.3, range = 18–103 years) and 50% was female (see Table 1). About 67.1% of the sample was familiar with the r-number, and 42.1% was highly numerate by answering all three numeracy items correctly. Participants were comparable in terms of sociodemographics and objective numeracy across all four experimental conditions.

**Table 1:** Participant characteristics (n *=* 1,168)

Characteristic	*n*	%
**Gender**		
Female	587	50.3
Male	581	49.7
**Age, mean (SD)**	55.6	17.3
18–24 years	55	4.7
25–34 years	126	10.8
35–44 years	131	11.2
45–54 years	207	17.7
55–64	225	19.3
≥ 65 years	424	36.3
**Education** ^**a**^		
Low (primary and low levels of secondary school)	328	28.1
Medium (higher levels of secondary school and practical education)	394	33.7
High (college and university)	445	38.1
**Objective numeracy**		
Less numerate	676	57.9
Highly numerate	492	42.1

Note.^a^ = 1 missing value

### Objective understanding of the r-number

In general, 650 participants (55.7%) correctly understood the r-number (i.e., giving “138” as their answer), and when comparing the four experimental conditions there were no differences, χ^2^(3, 1168) = 6.11, *P =* .106 (Table 2). Communicating the r-number with a visual aid (% correct: 55.3%) did not lead to a better understanding than without a visual aid (% correct: 56%), χ^2^(1, 1168) = 0.62, *P =* .803. Examples of alternative (yet incorrect) answers given by participants who saw the visual aid included “38” (4.9% of answers), “1,380” (3.5% of answers), or “200” (1.7% of answers). For those who did not see a visual aid, participants also provided incorrect answers such as “38” (8.4% of answers), “1,380” (2.1% of answers), or “200” (1.6% of answers).

**Fig. 3:**
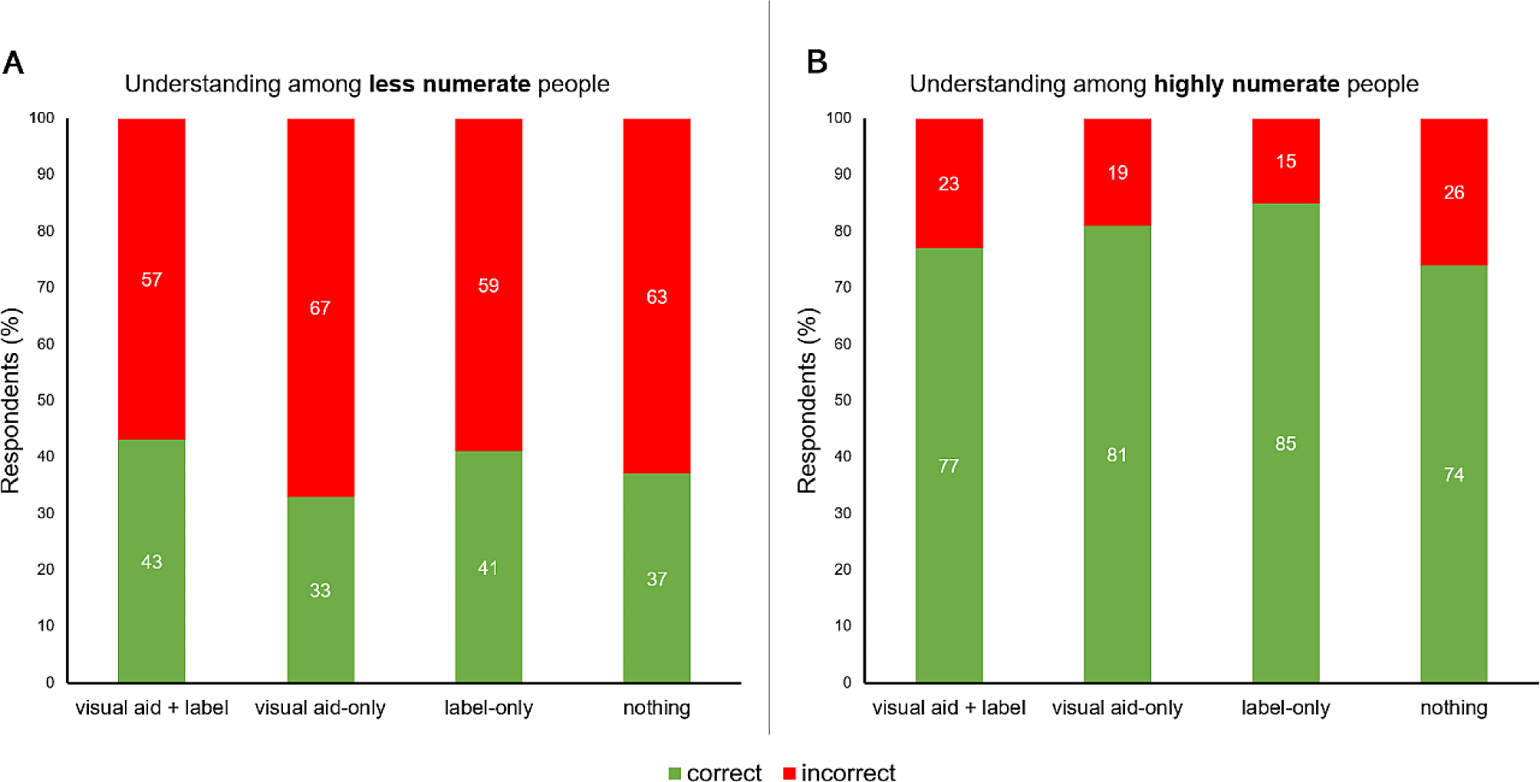
Objective understanding of the COVID-19 r-number as a function of experimental condition and numeracy

Those receiving the r-number with an evaluative label had a better understanding (% correct: 59.2%) than those without an evaluative label (% correct: 52.2%), χ^2^(1, 1168) = 5.74, *P =* .017. Highly numerate people (% correct: 79.1%) also had a better understanding of the r-number than less numerate people (% correct: 38.6%), χ^2^(1, 1168) = 188.83, *P* < .001, but none of the experimental conditions affected understanding among both highly (χ^2^(1, 492) = 4.73, *P =* .193) and less (χ^2^(1, 676) = 4.75, *P =* .191) numerate people (Fig. 3).

### Evaluation of the r-number

Descriptive statistics of the three outcome measures for people’s affective and cognitive evaluations, and their perceived usefulness of the r-number are shown in Table 2, and the complete statistics associated with the three separate factorial ANOVAs are displayed in Table 3. People’s affective evaluation of the r-number was not influenced by the inclusion or exclusion of an evaluative label, *F*(1, 1157) = 1.11, *P* = .293, η^2^ = 0.001, or with or without a visual aid, *F*(1, 1157) = 2.14, *P* = .144, η^2^ = 0.002. There was also no statistically significant interaction effect between evaluative label and visual aid on people’s affective evaluation, *F*(1, 1157) = 0.95, *P* = .332, η^2^ = 0.001, nor was there an interaction with numeracy, *F*(1, 1157) < 1, *P* = .505, η^2^ = 0.000.

Similarly, people’s cognitive evaluation of the r-number was not influenced by the presence of an evaluative label, *F*(1, 1157) < 1, *P* = .571, η^2^ = 0.000, and/or a visual aid *F*(1, 1157) < 1, *P* = .748, η^2^ = 0.000, nor was there a statistically significant interaction between the two factors *F*(1, 1157) < 1, *P* = .532, η^2^ = 0.000. However, regardless of format, highly numerate people found the r-number more simple, clearer, and easier to understand (*M* = 3.97, *SD* = 0.64) than less numerate people (*M* = 3.59, *SD* = 0.73), *F*(1, 1157) = 84.41, *P* < .001, η^2^ = 0.068.

Furthermore, information about the r-number was perceived as more useful when being presented with a visual aid (*M* = 3.68, *SD* = 0.71) than without (*M* = 3.59, *SD* = 0.72), *F*(1, 1158) = 4.34, *P* = .038, η^2^ = 0.004. Moreover, regardless of format, highly numerate people (*M* = 3.69, *SD* = 0.71) also perceived the r-number as more useful than less numerate people (*M* = 3.60, *SD* = 0.72), *F*(1, 1158) = 5.33, *P* = .021, η^2^ = 0.005.

**Table 2:** Participants’ scores (with standard deviations within parentheses) on understanding, affective evaluation, cognitive evaluation, and perceived usefulness as a function of evaluative label and visual aid

	Evaluative label			No evaluative label			Total	
	Visual aid (*N* = 300)	No visual aid (*N =* 278)	Total	Visual aid (*N =* 295)	No visual aid (*N =* 293)	Total	Visual aid	No visual aid
**Understanding (% correct)**	58.0	60.4	59.2	52.5	51.9	52.2	55.3	56.2
**Cognitive evaluation**	3.77 (0.66)	3.74 (0.73)	3.76 (0.70)	3.75 (0.77)	3.76 (0.71)	3.75 (0.74)	3.76 (0.72)	3.75 (0.76)
**Affective evaluation**	3.37 (0.81)	3.36 (0.93)	3.37 (0.87)	3.38 (0.87)	3.25 (0.91)	3.31 (0.89)	3.38 (0.84)	3.31 (0.92)
**Perceived usefulness**	3.67 (0.70)	3.61 (0.73)	3.64 (0.71)	3.68 (0.72)	3.58 (0.71)	3.63 (0.72)	3.68 (0.71)	3.60 (0.72)

**Table 3:** Main and interaction effects on people’s evaluation of the r-number (i.e., affective evaluation, cognitive evaluation, and perceived usefulness) resulting from three separate 2 (evaluative label) × 2 (visual aid) × 2 (numeracy) ANOVAs.

	Affective evaluation^a^			Cognitive evaluation^a^			Perceived usefulness^b^		
Main/Interaction Effects	F	*p*	η_*p*_^2^	F	*p*	η_*p*_^2^	F	*p*	η_*p*_^2^
Evaluative label	1.11	0.293	0.001	< 1	0.571	0.000	< 1	0.916	0.000
Visual aid	2.14	0.144	0.002	< 1	0.748	0.000	**4.34**	**0.038**	**0.004**
Numeracy	< 1	0.756	0.000	**84.42**	**< 0.001**	**0.068**	**5.33**	**0.021**	**0.005**
Evaluative label × Visual aid	< 1	0.332	0.001	< 1	0.532	0.000	< 1	0.671	0.000
Visual aid × Numeracy	< 1	0.606	0.000	< 1	0.434	0.001	< 1	0.529	0.000
Evaluative label × Numeracy	< 1	0.580	0.000	< 1	0.880	0.000	< 1	0.889	0.000
Evaluative label × Visual aid × Numeracy	< 1	0.505	0.000	< 1	0.933	0.000	< 1	0.670	0.000

*Note.*^a^*df* = 1, 1157; ^b^*df* = 1, 1158; ^c^ Significant results are given in bold

### Changes in adherence and perceived susceptibility after exposure to the r-number

**Fig. 4:**
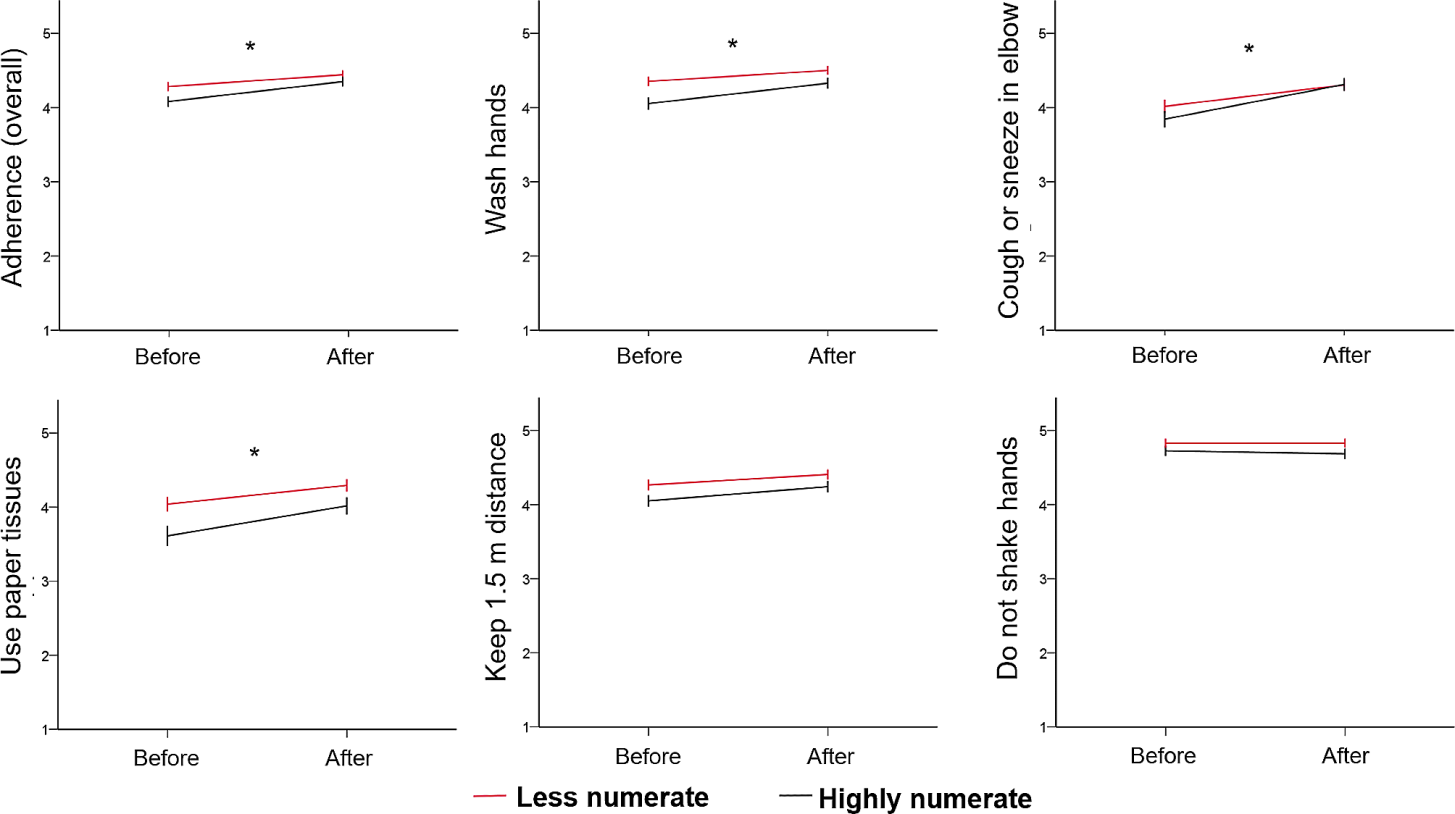
Adherence to preventive measures in the past seven days before exposure to the r-number and intention to adhere in the upcoming month after exposure to the r-number, separated for less and highly numerate people. Note: * refers to the significant interaction effect between time (before and after) and numeracy (less and highly numerate people), with *P* < .01

Compared to baseline adherence scores (*M* = 4.19, *SD* = 0.70), the intention to adhere to preventive measures increased after seeing the r-number (*M* = 4.40, *SD* = 0.67), *F*(1, 1156) = 290.79, *P* < .001, η^2^ = 0.201. These changes were not influenced by the presence of an evaluative label, *F*(1, 1156) < 1, *P* = .873, η^2^ = 0.005, or a visual aid, *F*(1, 1156) < 1, *P* = .863, η^2^ = 0.000. However, there was a statistically significant interaction between measurement (pre/post adherence) and numeracy, *F*(1, 1156) = 19.71, *P* < .001, η^2^ = 0.017, with only highly numerate people reporting significant changes in their intention to adhere after being exposed to the r-number (adherence before exposure: *M* = 4.08, *SD* = 0.68 vs. intention to adhere after exposure: *M* = 4.35, *SD* = 0.62), irrespective of the format. These changes were not observed in less numerate people (adherence before exposure: *M* = 4.28, *SD* = 0.79 vs. intention to adhere after exposure: *M* = 4.44, *SD* = 0.70). As shown in Fig. 4, these changes in adherence after exposure to the r-number among highly numerate people were found for the measures “washing hands”, *F*(1, 1156) = 14.48, *P* < .001, η^2^ = 0.012, “sneezing in elbow”, *F*(1, 1156) = 11.64, *P* < .001, η^2^ = 0.010, and “using paper tissues”, *F*(1, 1156) = 8.82, *P* = .003, η^2^ = 0.008, but not for “keeping 1.5 meter distance”, *F*(1, 1156) = 1.55, *P* = .214, η^2^ = 0.001, or “not shaking hands”, *F*(1, 1156) = 2.23, *P* = .136, η^2^ = 0.002.

Compared to baseline scores (*M* = 3.09, *SD* = 0.65), perceived susceptibility to COVID-19 was not higher after seeing the r-number (*M* = 3.07, *SD* = 0.68), *F*(1, 1156) = 2.02, *P* = .155, η^2^ = 0.002, regardless of an evaluative label, *F*(1, 1156) < 1, *P* = .429, η^2^ = 0.001, a visual aid, *F*(1, 1156) = 1.70, *P* = .192, η^2^ = 0.001, or numeracy, *F*(1, 1156) < 1, *P* = .530, η^2^ = 0.000.

## Discussion

In times of major infectious disease outbreaks, epidemiologists and policy makers often collect large amounts of quantitative data about transmission and subsequently share these with the general public. Yet, people do not always understand these complex numbers, let alone use them and act upon them [2, 12]. In this experimental study among a large Dutch representative sample, we focused on the COVID-19 basic r-number and examined how people with different numeracy skills would understand, evaluate, and use the number depending on how it is presented to them.

The findings suggest that people, especially those with lower numeracy skills, find understanding the r-number for infectious diseases quite difficult. This limited objective understanding is an important finding, as the r-number was one of the most important statistics being shared with the general public during the COVID-19 pandemic [1, 2, 8]. Taking dual coding and cognitive load theory as a starting point [26], we expected that understanding of the r-number could be increased by presenting the statistical information both verbally (i.e., written words) and visually (i.e., by adding an icon-based tree diagram for showing how the r-number works), particularly among less numerate people [58]. Although the visual aid was perceived as useful (compared to a verbal-only format), adding a visual aid to the verbal information did not lead to a better objective understanding of the numerical concept among both highly and less numerate people. A possible explanation for this is that the tree diagram used in the current study only showed the exponential growth of a basic r-number of 0, 1, and 2 in three reproductive stages. People therefore still needed to do the math themselves to understand the r-number used in the current study (“1.38”), which was arguably challenging for less numerate people. Speculatively, the visual aid could have helped people in getting better understanding of the main idea behind the r-number in light of concepts such as exponential growth of more infected cases in, but we did not test this “gist understanding” (a concept derived from fuzzy-trace theory [59]) in our experiment.

When the r-number was explained with the visual aid, the information on the dashboard was evaluated as more useful. However, regardless of whether the r-number was presented with or without evaluative labels and visual aids, highly numerate people evaluated the information on the dashboard as more simple, clear, and easy to understand compared to less numerate people. Furthermore, communicating the r-number with an evaluative label in the shape of a categorical line display did not lead to differences in people’s affective evaluation of the information (i.e., not having any impact on how serious or frightening people found the information was). Although previous research has shown that evaluative labels may lead to unwanted higher affective evaluations or emotional responses (e.g., fear or worry) [43, 46], we did not observe such “emotional” side-effects when using color-based categorical line displays.

Our results show that the r-number may be used to motivate people to (even) better adhere to certain preventive measures to limit the spread of the virus, although evaluative labels and visual aids did not impact this. After being exposed to the r-number, we noticed a slight increase people’s intention to adhere to protective measures such as “washing hands”, “sneezing in elbow”, and “using paper tissues” in the upcoming month. Interestingly, this significant change was only shown for highly numerate people, who also demonstrated the highest levels of understanding. But, importantly, for at least for part of the population, providing numbers is beneficial, as it motivates them to engage in healthier and protective behavior [10, 17]. Speculatively, differences in cognitive elaboration and numeric sensitivity between highly and less numerate individuals could explain this effect. Even though both numeracy groups received numerical information about the r-number, highly numerate are known to be more numerically sensitive [17], and they could therefore have spent more time looking at the r-number compared to the less numerate. This enhanced cognitive elaboration on the r-number could have led highly numerate participants to better understand and evaluate the situation, hence leading to increased adherence scores. However, as we did not collect any behavioral data about where people looked at during the experiment (e.g., obtained through eye-tracking), we can only speculate about this. Future research is needed to provide empirical evidence for theoretical predictions about how people with various numeracy skills cognitively process and behaviorally act upon pandemic-related statistics, such as the r-number, in times of crises [9].

A strength of this study is that we used real-world and actual numerical data about the r-number (through the official Dutch corona dashboard [5]) that was accurate and relevant in the period of data collection (i.e., October 2020), which boosted the ecological validity of the study. However, a potential limitation is that we only tested understanding of a single r-number in a specific period in the corona pandemic, which limits the generalizability of our findings to other r-numbers. There is now strong evidence suggesting that people’s risk perceptions of COVID-19 and adherence to preventive measures may change over time [60], and may depend on specific milestones in the pandemic such as the onset of the crisis, the implementation and relaxation of protective measures, or – in the case of this study – on the occurrence of another “wave” with a significant rise in the number of infected cases and hospital admissions rates. As such, we cannot draw any conclusions about how people perceive and respond to r-numbers that are equal or below 1, or to the r-number in times of relaxations of government measures, although we conjecture that the impact of such changing factors on understanding is relatively small. Similarly, we assessed adherence to preventive measures either by relying on self-reported adherence in the past seven days (before exposure to the r-number) and intention to adhere in the upcoming month (after exposure to the r-number), but real behavioral data on these outcomes is lacking.

Similar to the evaluative label, the visual aid that was used in the experiment was taken from the official Dutch corona dashboard. Given the nature of this visual aid (which visualizes how the r-number generally works for *r* = 0, *r* = 1.0, and *r* = 2.0), it could have been challenging for people to apply the visual aid to the r-number in the current experiment (i.e., *r* = 1.38). For instance, participants could have mistakenly thought that an r-number of 1.38 means that 100 people would infect 200 people (instead of 138 people). Nonetheless, when observing participants’ answers to the objective understanding item, we only noticed a small proportion of such answers in both the experimental condition with and without a visual aid (1.7% and 1.6% of the answers, respectively). Nevertheless, more systematic research is needed to further test the effects of other types of visual aids combined with various r-values on people’s understanding, emotional responses, and behavior.

Although we used a valid scale for measuring people’s objective numeracy skills [57], we did not measure other relevant individual difference factors such as graph literacy (i.e., the ability to understand and extract data and meaning from visual formats) [30]. Studies have suggested that less numerate people may benefit from visual aids (e.g., pictographs), but especially when having high graph literacy skills [20, 58]. In fact, there are many other individual difference factors (e.g., uncertainty tolerance or information coping style) that may moderate the effects of different risk communication formats on people’s understanding of complex numerical health data. In addition, the visual materials in our experiment were assumed to be simple, but they did not fully include clear textual explanations for conveying the bottom-line meaning of the current r-number. However, we cannot draw conclusions about whether the evaluative label improved people’s gist knowledge (i.e., getting the bottom-line meaning of the message), since we the aim of our study was to measure objective (mis)understanding of the r-number (i.e., correct vs. incorrect answer).

Our findings provide valuable guidance to public health communicators, policy makers, and those presenting public health and scientific data related to pandemic outbreaks via web-based dashboards [5–7]. Our results call for testing other strategies for effectively communicating the r-number to the general public and to make such statistics more meaningful, especially for people who experience difficulties in using and drawing meaning from numbers. Less numerate people may not be able to fully get the gist of statistical information or may even ignore numerical data [17]. If people do not have the capacity to fully understand these statistics, they are less likely to act and change their behavior [14, 15]. Therefore, telling people explicitly what the r-number means (e.g., directly telling patients whether the number is alarming or not) but at the same time also informing them about any concrete actions that they can take themselves (e.g., providing simple yet effective solutions or measures that people believe they can take), may help people with various numeric abilities to translate complex public health data into meaningful and appropriate actions [9, 10].

## Conclusions

Our findings show that people (especially less numerate people) find the task to understand the r-number for infectious diseases difficult. Although we found no evidence for the facilitating role of evaluative labels and visual aids on people’s understanding of the r-number, our results do suggest that the statistic may be used to stimulate people to better adhere to certain preventive measures to limit the spread of infectious diseases. Policy makers and public health communicators are advised to clearly explain why they are giving these numbers to people, but also what people should do with them to stimulate behavior change in combatting the spread of virus during a pandemic.

## Data Availability

The datasets generated during and/or analyzed during the current study and the complete online experiment (with materials and measure items) are available in the Open Science Framework repository, https://osf.io/mxqhz/ [49].

## Abbreviations

ANOVA: Analysis of variance
r-number: Reproduction number
